# Metabolic engineering of *Bacillus subtilis* for redistributing the carbon flux to 2,3-butanediol by manipulating NADH levels

**DOI:** 10.1186/s13068-015-0320-1

**Published:** 2015-08-27

**Authors:** Taowei Yang, Zhiming Rao, Guiyuan Hu, Xian Zhang, Mei Liu, Yue Dai, Meijuan Xu, Zhenghong Xu, Shang-Tian Yang

**Affiliations:** The Key Laboratory of Industrial Biotechnology, Ministry of Education, School of Biotechnology, Jiangnan University, Wuxi, 214122 Jiangsu People’s Republic of China; Laboratory of Pharmaceutical Engineering, School of Pharmaceutical Science, Jiangnan University, Wuxi, 214122 Jiangsu People’s Republic of China; Department of Chemical and Biomolecular Engineering, The Ohio State University, Columbus, OH 43210 USA; School of Biotechnology, Jiangnan University, 1800 Lihu Avenue, Wuxi, 214122 Jiangsu People’s Republic of China

**Keywords:** 2,3-Butanediol, Manipulating NADH levels, Disruption, *Bacillus subtilis*

## Abstract

**Background:**

Acetoin reductase (Acr) catalyzes the conversion of acetoin to 2,3-butanediol (2,3-BD) with concomitant oxidation of NADH to NAD^+^. Therefore, intracellular 2,3-BD production is likely governed by the quantities of rate-limiting factor(s) Acr and/or NADH. Previously, we showed that a high level of Acr was beneficial for 2,3-BD accumulation.

**Results:**

Metabolic engineering strategies were proposed to redistribute carbon flux to 2,3-BD by manipulating NADH levels. The disruption of NADH oxidase (YodC, encoded by *yodC*) by insertion of a formate dehydrogenase gene in *Bacillussubtilis* was more efficient for enhancing 2,3-BD production and decreasing acetoin formation than the disruption of YodC by the insertion of a Cat expression cassette. This was because the former resulted in the recombinant strain AFY in which an extra NADH regeneration system was introduced and NADH oxidase was disrupted simultaneously. On fermentation by strain AFY, the highest 2,3-BD concentration increased by 19.9 % while the acetoin titer decreased by 71.9 %, relative to the parental strain. However, the concentration of lactate, the main byproduct, increased by 47.2 %. To further improve carbon flux and NADH to 2,3-BD, the pathway to lactate was blocked using the insertional mutation technique to disrupt the lactate dehydrogenase gene *ldhA*. The resultant engineered strain *B. subtilis* AFYL could efficiently convert glucose into 2,3-BD with little acetoin and lactate accumulation.

**Conclusions:**

Through increasing the availability of NADH and decreasing the concentration of unwanted byproducts, this work demonstrates an important strategy in the metabolic engineering of 2,3-BD production by integrative recombinant hosts.

## Background

2,3-Butanediol (2,3-BD) is an important platform chemical due to its wide industrial applications [1–3]. Significantly, 2,3-BD is a potentially valuable fuel additive with a heating value of 27.2 kJ/g, comparable to that of other liquid fuels (e.g., ethanol 29.055 kJ/g, methanol 22.081 kJ/g) [4]. Because of the shortage of fossil fuels and the development of biorefineries from renewable resources, microbial production of 2,3-BD has attracted growing attention [1, 2].

A number of microorganism genera are employed to produce 2,3-BD, including *Klebsiella*, *Enterobacter*, *Bacillus*, and *Serratia* [5]. Among these strains, *B. subtilis*, generally regarded as safe (GRAS) [6], is often used as a platform organism to engineer Gram-positive bacterial pathways for industrial production of various secondary metabolites [7]. In such bacterial metabolism, glucose is first converted to pyruvate before generation of major products. In addition to 2,3-BD, the pyruvate produced from glucose is channeled into a mixture of ethanol, acetoin, formate, acetate, and lactate, through the mixed acid-2,3-BD fermentation pathway [1, 2, 5]. In previous studies, several strategies were used to improve 2,3-BD production, such as optimizing culture conditions, and establishing mathematical models [8–10].

**Fig. 1:**
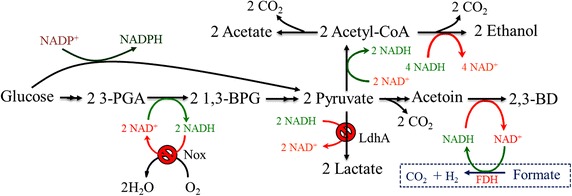
Metabolic engineering strategies for redistributing the carbon flux to 2,3-BD by manipulating NADH levels. 2,3-BD is an NADH-dependent product; the NADH availability and its proportion in the active form play important roles in 2,3-BD production. But NADH oxidase catalyzes the oxidation of NADH to NAD^+^. Lactate, one of the main by-products, competes with 2,3-BD for pyruvate as a metabolic intermediate and NADH as a cofactor. To block a pathway that competitively consumes NADH and to redirect carbon flux towards 2,3-BDO production, the NADH oxidase was disrupted by insertion of a formate dehydrogenase gene *fdh*, and then the pathway to lactate was blocked using the insertional mutation technique to disrupt the lactate dehydrogenase gene *ldhA*. (*3-PGA* 3-Phosphate glyceraldehyde, *1,3-BPG* 1,3-Bisphosphoglycerate, *Red arrows*, oxidation of NADH to NAD^+^; *Green arrows*, reduction of NAD^+^ to NADH; , blocking the pathway; The *blue dotted box*, introducing extrinsic pathway)

The 2,3-BD pathway takes part in regulating the intracellular NAD^+^/NADH ratio [1, 2]. Because 2,3-BD, ethanol, and lactic acid are NADH-dependent products involved in the 2,3-BD pathway (Fig. 1), NADH availability and its proportion in the active form (i.e. as NADH rather than NAD^+^) play a key role in dictating the entire process yield of 2,3-BD. 2,3-BD production could be further improved through manipulation of the NADH-dependent pathways. To block a pathway that competitively consumes NADH and to redirect carbon flux towards 2,3-BD production, many investigators [11–14] have attempted to construct different lactate dehydrogenase (LdhA; encoded by the *ldhA* gene) inactivated strains. They found that inactivation of LdhA resulted in improving 2,3-BD production, while suppressing lactate and acetoin formation.

Furthermore, Ji et al. [15] successfully constructed a *K. oxytoca* mutant deficient in ethanol accumulation by replacing the *aldA* gene (coding aldehyde dehydrogenase) with a tetracycline resistance cassette. They found that the carbon flux to 2,3-BD was improved by eliminating the ethanol pathway and in the meantime suppressing the formation of another main byproduct, acetoin. A double mutant *K. pneumoniae* strain with deletion of *adhE* (coding acetaldehyde dehydrogenase) and *ldhA* was constructed by Guo et al. [16], which resulted in accelerated fermentation and higher 2,3-BD production. Previously, we introduced extra copies of glyceraldehyde-3-phosphate dehydrogenase (GpdH) and acetoin reductase (Acr) into the GRAS strain *B. amyloliquefaciens* and found that overexpressing this NADH/NAD^+^ regeneration system effectively improved 2,3-BD production and inhibited byproduct accumulation [17]. Dai et al. [18] reported that the addition of vitamin C could elevate the NADH/NAD^+^ ratio, resulting in a remarkable increase of 2,3-BD titer in *Paenibacillus polymyxa* CJX518.

Acetoin reductase could convert acetoin to 2,3-BD, with concomitant oxidation of NADH to NAD^+^. Therefore, intracellular 2,3-BD production is likely governed by the quantities of the rate-limiting factor(s) the acetoin dehydrogenase complex AcoABCL (Acr plays the most important role) and/or NADH. Previously, we identified that low levels of Acr and NADH were key factors that limited acetoin degradation and found that increasing the Acr level in *B. subtilis* was beneficial for 2,3-BD production [17, 19], but there were still large quantities of acetoin and lactate in the culture broth. In this work, metabolic engineering strategies were proposed to redistribute the carbon flux to 2,3-BD by manipulating NADH levels (Fig. 1).

## Results

### Inactivation of NADH oxidase and its effect on 2,3-BD production in *Bacillus subtilis*

Because 2,3-BD is an NADH-dependent product, the NADH availability and its proportion in the reduced form play important roles in 2,3-BD production. NADH oxidase catalyzes the oxidation of NADH to NAD^+^. Previously, we successfully screened a water-forming NADH oxidase (YodC, encoded by *yodC*, gene ID: 939506) from *B. subtilis* 168 [20]. Therefore, to prevent NADH oxidation, *yodC* was disrupted by transforming the plasmid T-*yodC*::*cat* into *B. subtilis* strain ACR. PCR amplification results indicated that the *yodC* gene was successfully knocked out from *B. subtilis* ACR, and the activity of YodC was barely detectable in the resulting recombinant strain AY.

**Fig. 2:**
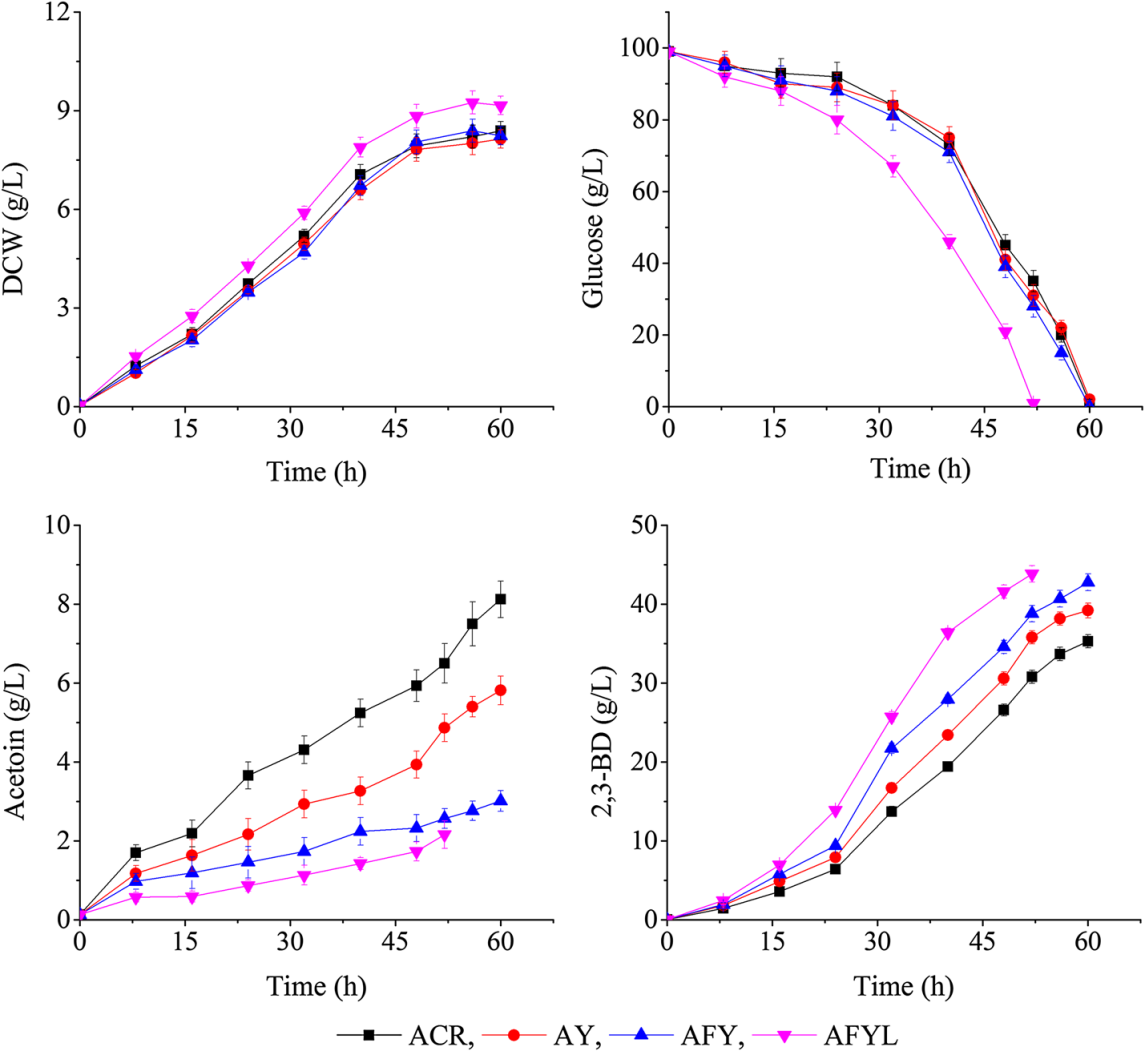
Time profiles of 2,3-BD fermentation with various strains. (ACR, *B. subtilis* 168 with pMA5-*acr*; AY, ACR blocked in YodC by insertion of a Cat expression cassette; AFY, Acr blocked in YodC by insertion of *fdh* gene; AFYL, AFY blocked in LdhA by insertion of a Cat expression cassette. All assays were performed by triplicate cultures, standard deviations of the biological replicates were represented by *error bars*)

**Fig. 3:**
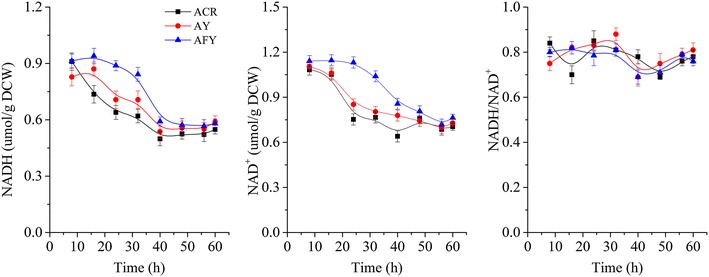
Time profiles of intracellular NADH and NAD^+^ concentrations of various strains. (ACR, *B. subtilis* 168 with pMA5-*acr*; AY, ACR blocked in YodC by insertion of a Cat expression cassette; AFY, Acr blocked in YodC by insertion of *fdh* gene; AFYL, AFY blocked in LdhA by insertion of a Cat expression cassette. All assays were performed by triplicate cultures, standard deviations of the biological replicates were represented by *error bars*)

The effects of disruption of YodC on cell growth and the intracellular concentrations of NADH and NAD^+^ were observed. Figure 2 shows that strain AY grew at a similar rate to the parental strain ACR, indicating that the disruption of YodC in *B. subtilis* ACR did not affect cell growth. In both the parental and engineered strains, intracellular concentrations of NADH, NAD^+^ and NAD^+^/NADH ratio continuously changed during the batch fermentation, but no significant difference was observed between the two strains (Fig. 3). This suggests that the redox balance in strain AY was not disturbed, which also explains why cell growth of strain AY was not significantly affected (Fig. 2).

**Fig. 4:**
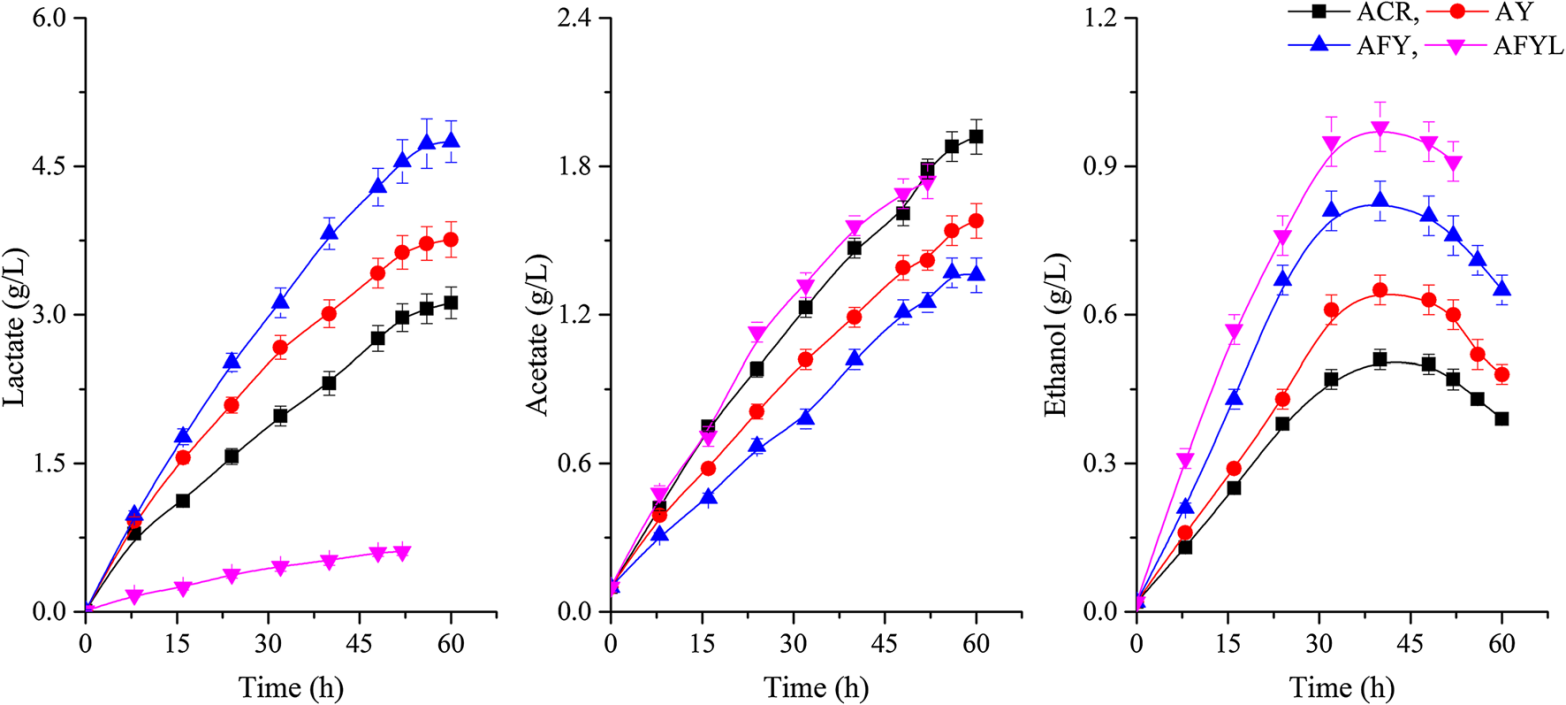
Time profiles of lactate, acetate, and ethanol production with various strains. (ACR, *B. subtilis* 168 with pMA5-*acr*; AY, ACR blocked in YodC by insertion of a Cat expression cassette; AFY, Acr blocked in YodC by insertion of *fdh* gene; AFYL, AFY blocked in LdhA by insertion of a Cat expression cassette. All assays were performed by triplicate cultures: standard deviations of the biological replicates were represented by *error bars*)

The effects of disruption of YodC on 2,3-BD production were also observed. Figure 2 shows that the glucose consumption rates were very similar between the parental and engineered strains. 2,3-BD production was enhanced in strain AY (Fig. 2), indicating that disruption of YodC increased NADH availability. The highest 2,3-BD concentration was increased by 8.8 % relative to the parental strain, while that of acetoin was decreased by 23.6 % (Fig. 2; Table 1). However, compared with the parental strain, the molar yield of the major unwanted byproduct lactate was 16.7 % higher in the engineered strain AY (Fig. 4; Table 1). This suggests that the ‘‘extra’’ NADH generated by inactivating YodC was utilized in other NADH-dependent pathways (especially in 2,3-BD, lactate pathways), which explains why the knockout of the yodC gene has no significant influence on the NAD +/NADH ratio.

### Disruption of NADH oxidase gene *yodC* and simultaneous introduction of an extra NADH regeneration system in *B. subtilis*

Although disruption of YodC increased the 2,3-BD yield, the acetoin accumulation in strain AY remained significant. Heterogeneous production of formate dehydrogenase (Fdh, encoded by *fdh*) in *B. subtilis* was expected to increase the overall intracellular NADH pool, thereby improving the flux through NADH-dependent pathways (mainly the 2,3-BD pathway). The plasmid T-*yodC*::f*dh* was transformed into *B. subtilis* ACR, resulting in recombinant strain AFY in which an extra NADH regeneration system was introduced and YodC was simultaneously disrupted. PCR amplification results indicated that the *yodC* gene was successfully knocked out in strain AFY. The Fdh activity was increased by about 15-fold relative to the parental strain. To use the NADH regeneration capability of Fdh, extra sodium formate was needed in the culture medium so that the reaction NaCOOH + NAD^+^ → CO_2_ + NADH + Na^+^ could be catalyzed by Fdh [21]. We found that cell growth of strain AFY was not affected when the concentration of sodium formate was lower than 40 mM (data not shown). Based on the three-stage division of cell growth curves (Fig. 2), 30 mM sodium formate was fed into the cultivation broth of strain AFY at 12, 24, and 48 h after culture inoculation, respectively, to serve as the substrate in the reaction catalyzed by Fdh.

Production of Fdh in *B. subtilis* AFY led to a slower decrease of both NADH and NAD^+^ pools than was observed for the parental strain. Meanwhile, there was no remarkable difference in the ratio of NADH/NAD^+^ between the parental and engineered strains (Fig. 3). These data suggest that the redox balance in *B. subtilis* AFY producing Fdh was not disturbed, as also observed in *K. oxytoca* by Zhang et al. [21], which explains why the cell growth of strain AFY was not remarkably affected (Fig. 2). Furthermore, the consumption rate of glucose was not notable altered between the engineered strain AFY and the parental strain Acr (Fig. 2). 2,3-BD was the main product of strain AFY metabolism, followed by lactate and acetoin (Table 1). The maximum 2,3-BD concentration increased by 18.5 % and the acetoin titer decreased by 65.7 % in engineered strain AFY relative to the parental strain (Fig. 2; Table 1). However, the concentration of the main byproduct, lactate, increased by 47.2 % in batch fermentation compared with the corresponding level in strain ACR.

### Enhanced 2,3-BD yield through inactivation of lactate dehydrogenase in *B. subtilis* AFY

Disruption of the NADH oxidase gene and simultaneous introduction of an extra NADH regeneration system in *B. subtilis* not only enhanced 2,3-BD production and suppressed acetoin accumulation, but also increased byproduct lactate production, which negatively regulates the 2,3-BD yield and increases the costs of downstream separation and purification. Therefore, we also knocked out LDH (gene *ldhA*) by transforming plasmid T-*ldhA*::*cat* into *B. subtilis* AFY. The PCR amplification results indicated the *ldhA* gene was successfully knocked out from *B. subtilis* AFYL, and the activity of LdhA decreased by 90.1 %, which confirmed that *ldhA* was the major LdhA-producing enzyme in *B. subtilis*.

The inactivation of LdhA resulted in faster cell growth and shorter fermentation time than for the parental and AFY strains (Fig. 2). Similar stimulatory effects on cell growth and glucose consumption were also observed by Guo et al. [16] in an analogous *K. pneumoniae* strain. Furthermore, as shown in Fig. 2 and Table 1, the maximum 2,3-BD concentration increased by 25.5 %, while the acetoin titer decreased by 76.4 %, in engineered strain AFYL relative to strain ACR; the lactate concentration decreased by 81.1 % (Fig. 4; Table 1).

**Table 1:** The glucose metabolic flux distribution in different *B. subtilis* strains (unit: mol/mol glucose)

Strain	2,3-BD	Acetoin	Lactate	Ethanol	Acetate	Biomass^a^
ACR	0.702 ± 0.021	0.178 ± 0.005	0.036 ± 0.001	0.015 ± 0.001	0.032 ± 0.001	0.042 ± 0.002
AY	0.764 ± 0.022	0.136 ± 0.004	0.042 ± 0.002	0.016 ± 0.001	0.026 ± 0.001	0.042 ± 0.002
AFY	0.832 ± 0.024	0.061 ± 0.003	0.053 ± 0.002	0.018 ± 0.001	0.023 ± 0.001	0.041 ± 0.002
AFYL	0.881 ± 0.025	0.042 ± 0.002	0.007 ± 0.000	0.021 ± 0.001	0.029 ± 0.001	0.046 ± 0.002

All assays were performed by triplicate cultures; standard deviations of the biological replicates were represented by error bars

ACR, *B. subtilis* 168 with pMA5-*acr*; AY, ACR blocked in YodC by insertion of a Cat expression cassette; AFY, Acr blocked in YodC by insertion of *fdh* gene; AFYL, AFY blocked in LdhA by insertion of a Cat expression cassette

^a^Assuming the biomass formula is C_3.93_H_7.07_O_1.96_N_0.79_ with 3.35 % ash [34]

**Table 2:** Strains, plasmids, and primers used in this study

Bacterial strain, plasmid or primer	Relevant characteristic or sequence	Source or restriction
Strains		
*B. subtilis* ACR	*B. subtilis* 168 with pMA5-HpaII-*acr*, source of *yodC* and *ldhA* genes	Lab stock
*B. subtilis* AY	*B. subtilis* ACR blocked in YodC by insertion of a Cat expression cassette	This study
*B. subtilis* AFY	*B. subtilis* ACR blocked in YodC by insertion of *fdh* gene	This study
*B. subtilis* AFYL	*B. subtilis* AFY blocked in LdhA by insertion of a CAT expression cassette	This study
*E. coli* JM109	recA1, endA1, gyrA96, thi-1, hsd R17(rk- mk +)supE44	Invitrogen
*Candida boidinii*	Source of *fdh* gene	Lab stock
Plasmids		
pMD18-T	Cloning vector	Takara
pBGSC6	Containing Cat expression cassette	Lab stock
pMA5-HpaII	Expression vector (in *E. coli*, Ap^r^; in *B. subtilis*, Kan^r^)	Lab stock
T-*cat*	pMD18-T with Cat expression cassette	This study
T-*yodC*	pMD18-T with *yodC*	This study
T-*yodC*::*cat*	*cat* inserted into the *Eco*R V site of T-*yodC*	This study
T-*fdh*	pMD18-T with *fdh*	This study
pMA5-*fdh*	pMA5-HpaII with *fdh*	
T-HpaII-*fdh*	pMD18-T with HpaII-*fdh*	This study
T-*yodC*::*fdh*	*fdh* inserted into the *Eco47* III site of T-*yodC*	This study
T-*ldhA*	pMD18-T with *ldhA*	This study
T-*ldhA*::*cat*	*cat* inserted into the *Eco*R V site of T-*ldhA*	This study
Primers 5′-3′		
P1	ACCGGGATCCATGACGAATACTCTGGAT	*Bam*H I
P2	ACCGACGCGTTTACAGCCAAGTTGATAC	*Mlu* I
P3	CGCGATATCAAAAAAGGATTGATTCTAATG	*Eco*R V
P4	CGCGATATCTAGTGACATTAGAAAACCGAC	*Eco*R V
P5	CGCCATATGATGAAGATCGTTTTAGTC	*Nde* I
P6	CGCACGCGTTTATTTCTTATCGTGTTTAC	*Mlu* I
P7	ACCGAGCGCTATTTTTTGAGTGATCTTCTC	*Eco47* III
P8	ACCGAGCGCTTTATTTCTTATCGTGTTTAC	*Eco47* III
P9	ACCGGGATCCATGATGAACAAACATG	*Bam*H I
P10	ACCGACGCGTTTAGTTGACTTTTTGTTCTG	*Mlu* I

Underlined nucleotides are the restriction enzyme sites

## Discussion

In the 2,3-butanediol metabolic pathway, ACR catalyzes the conversion of acetoin to 2,3-BD with concomitant oxidation of NADH to NAD^+^ [7]. NADH oxidase (Nox) catalyzes the oxidation of NADH to NAD^+^ using molecular oxygen as the electron acceptor. Zhang et al. [20] identified an water-forming NADH oxidase (YodC) in *B. subtilis*. When YodC was over-expressed in *B. subtilis*, acetoin production was increased, while the production of 2,3-BD was decreased significantly. Ji et al. [22] and Sun et al. [23] also found that overexpression of NADH oxidase in *Klebsiella pneumoniae * resulted in a large increase of carbon flux to acetoin and in the ratio of acetoin to 2,3-BD. Because NADH is preferentially used in 2,3-BD synthesis, low levels of this coenzyme may limit the Acr reaction [17, 19]. Therefore, decreasing YodC activity which is possibly beneficial for 2,3-BD production, because NADH oxidase pathway competes with 2,3-BD branch for NADH as a cofactor. Inspired by this idea, we initially disrupted YodC in *B. subtilis* strain ACR, which increased not only the 2,3-BD yield, but also lactate and ethanol titer. It may be that disruption of YodC decreased metabolism of NADH and increased NADH availability, which resulted in increased fluxes to NADH-dependent pathways (e.g., 2,3-BD, lactate, and ethanol production pathways).

The fluxes through NADH-dependent pathways are determined by the availability of NADH, which could be improved by either decreasing the metabolic activity of branches competing for NADH [24, 25], or by introducing a NADH regeneration system [21]. The most successful NADH regeneration system involves the NAD^+^-dependent formate dehydrogenase gene *fdh* from *C. boidinii* [26]. Ma et al. [27] and Zhang et al. [21] found that introduction of this NADH regeneration system into *Klebsiella* sp. could efficiently improve glycerol metabolism and promote 1,3-propanediol yield. Therefore, we tried to simultaneously disrupt a NADH metabolic branch and introduce an extra NADH regeneration system into *B. subtilis*, and observe their effects on 2,3-BD metabolism. As expected, 2,3-BD production was further improved and acetoin accumulation was suppressed, relative to the parental strain. However, the production of byproducts lactate and ethanol was also enhanced; in particular, the lactate concentration was 47 % higher than for the parental strain. If disruption of YodC and introduction of an extra NADH regeneration system indeed increase the available NADH, all of the NADH-dependent pathways, (2,3-BD, lactate, and ethanol production pathways) would benefit from the enhancement.

Lactate is produced as a main byproduct during *B. subtilis* fermentation. Decreasing the production of this byproduct can be beneficial for 2,3-BD production, because lactate competes with 2,3-BD for pyruvate as a metabolic intermediate and NADH as a cofactor. Jung et al. [28] found that deleting the *ldhA* gene from *Enterobacter aerogenes* increased 2,3-BD production. In this study, we successfully deleted the *ldhA* gene from *B. subtilis* strain AFY to construct the recombinant strain AFYL. As expected, deletion of *ldhA* increased 2,3-BD production, by 25.5 %, and decreased the acetoin titer, by 76.4 %; significantly, the concentration of the principal byproduct lactate decreased by 81.1 % (all relative to strain ACR). These results for strain AFYL can be explained by the NADH consumption [12]: by blocking the lactate production pathway, the cell needed to consume NADH through other pathways; thus, the carbon flux toward the 2,3-BD pathway, and hence 2,3-BD biosynthesis, increased. More interestingly, the deletion of *ldhA* resulted in an increase in microbial cell growth. A similar stimulatory effect on cell growth was observed by Guo et al. [16] and Kim et al. [12] in *K. pneumoniae* and Jung et al. [28] in *E. aerogenes*. Generally, a mutant strain has a reduced growth rate compared to its parental strain. The main reason for the improved cell growth of the mutant could be that decreasing lactate production lowered the acidification rate of the culture medium [28].

## Conclusion

The GRAS engineered strain *B. subtilis* AFYL with *yodC* and *ldhA* deletion and *fdh* overexpression was constructed to redistribute carbon flux towards 2,3-BD biosynthesis by manipulation of the NADH levels. Strain AFYL exhibited elevated 2,3-BD production and reduced lactate and acetoin production. In this study, we demonstrated the construction of a target microorganism with its carbon flux redistributed towards 2,3-BD production through metabolic engineering.

## Materials and methods

### Bacterial strains, primers, and plasmids

All bacterial strains, primers, and plasmids used and constructed in this study are described in Table 2.

### Culture conditions

*Bacillus subtilis* and *Escherichia coli* were cultured in Luria–Bertani medium at 37 °C on a rotary shaker at 180 rpm. When necessary, 100 mg/L ampicillin, 25 mg/L chloromycetin, 50 mg/L kanamycin, or 900 mg/L formate [29] was added into the culture medium. Well-grown cells were selected for further positive colony selection.

For 2,3-BD fermentation, the seed culture was agitated in 250 mL shaken flasks for 10 h at 180 rpm and 37 °C. Then 1 mL seed culture (OD_600_ = 4.0–5.0) was inoculated into fermentation medium at 180 rpm and 37 °C. Seed culture medium contained 40 g/L glucose, 10 g/L peptone, 5 g/L yeast extract, and 10 g/L NaCl. The medium was sterilized at 121 °C for 20 min. The 2,3-BD fermentation medium (pH 6.5) contained 100 g/L glucose, 25 g/L corn steep liquor, 5 g/L ammonium citrate, 3 g/L K_2_HPO_4_, 0.6 g/L succinic acid, and 0.3 g/L MgSO_4_·7H_2_O. For preparing the 2,3-BD fermentation medium, glucose and the other supplements were separately sterilized at 121 °C for 20 min, and then the two solutions were mixed.

### Disruption of the *yodC* and *ldhA* genes

For disruption of the *yodC* gene, plasmids T-*yodC*::*cat* and T-*yodC*::*fdh* were constructed, respectively. The *yodC* gene encoding NADH oxidase (YodC) from *B. subtilis* strain ACR was amplified by PCR using primers P1 and P2 (Table 2). T-*yodC* was constructed by inserting *yodC* into the cloning sites of pMD-18T. The CAT expression cassette (encoded by *cat*) from the vector pBGSC6 was amplified using primers P3 and P4. The *fdh* gene encoding formate dehydrogenase (Fdh) from *Candida boidinii* was PCR amplified using primers P5 and P6. The amplified *fdh* gene was inserted into the *Nde* I and *Mlu* I sites of plasmid pMA5-HapII to create pMA5-*fdh*. The *fdh* gene plus the *Hap* II promoter from plasmid pMA5-*fdh* was then PCR amplified using primers P7 and P8. Then, *cat* and HapII-*fdh* were separately inserted into the *Eco47* III site of T-*yodC* to construct plasmids T-*yodC*::*cat* and T-*yodC*:: HapII-*fdh*. The plasmids T-*yodC*::*cat* and T-*yodC*:: HapII-*fdh* were transformed into *B. subtilis* ACR to construct the recombinant strains AY and AFY, respectively. The *ldhA* gene encoding LDH from *B. subtilis* ACR was amplified by PCR using primers P9 and P10. T-*ldhA* was constructed by inserting *yodC* into the cloning sites of pMD-18T. The *ldhA* gene was disrupted by the insertion of *cat* to construct plasmid T-*ldhA*::*cat*; then T-*ldhA*::*cat* was transformed into *B. subtilis* AFY to construct the recombinant strain AFYL. The insertions of the Cat expression cassette and HapII-*fdh* were confirmed by PCR. Plasmids were first transformed into *E. coli* JM109 cells by the calcium chloride method [18], subsequently isolated from *E. coli* JM109, and then transformed into *B. subtilis* using the method described by Vojcic et al. [30].

### Determination of NAD^+^ and NADH concentrations

The intracellular concentrations of NAD^+^ and NADH were determined by procedures described in our previous study [19].

### Enzyme assays

For preparation of crude YodC, the cell pellets collected by centrifugation were suspended and washed with 0.1 M potassium phosphate buffer (pH7.0) at least for three times. Then cells were resuspended in a washing buffer containing 0.15 mM FAD and 62.5 mM l-cysteine-HCl (PPFC-buffer). DNase (2000 units) and phenylmethylsulfonyl fluorid (1 mM) were then added to the suspension. The YodC activity was assayed as described by previous study by measuring the decrease of absorbance at 340 nm at 25 °C [31].

Cell-free extracts used for Fdh activity assay were prepared as previously described [21, 27]. Berríos-Rivera et al. [26] reported that Abs_340_ measurements are increased by cell extracts as NAD^+^ is reduced to NADH, which has an absorption maximum at 340 nm. From the resulting linear increase in Abs_340_, the Fdh activity can be measured. The reaction mixture (5 mL) contained 20 μL cell-free extract, 2.0 mM NAD^+^, 0.1 M sodium formate, and 100 mM 2-mercaptoethanol in phosphate buffer (10 mM, pH 7.5). The protein concentration of the cell-free extracts was determined by the method of Bradford [32]. All experiments were replicated at least three times.

### Analysis of cell growth and metabolites

The cell mass concentration was determined from the OD at 600 nm in an ultraviolet (UV)-visible spectrophotometer (UNICO UV-2000 spectrophotometer, Shanghai, China). The dry cell weight (DCW) could be calculated from OD_600_ using an equation that DCW (g/L) = 0.237 OD_600_ − 0.0126. The composition of the fermentation broth (glucose, 2,3-BD, acetoin, acetate, lactate, and ethanol) was determined by high-performance liquid chromatography [33]. All experiments were replicated at least three times.

## Abbreviations

2,3-BD: 2,3-butanediol
Acr: acetoin reductase
YodC: NADH oxidase
LdhA: lactate dehydrogenase
GRAS: generally regarded as safe
Fdh: formate dehydrogenase
